# Complete Genome Sequence of a New H9N2 Avian Influenza Virus Isolated in China

**DOI:** 10.1128/genomeA.00261-13

**Published:** 2013-05-30

**Authors:** Jing-Yu Wang, Juan-Juan Ren, Wan-Hua Liu, Pan Tang, Ning Wu, Chi-Young Wang, Ching-Dong Chang, Hung-Jen Liu

**Affiliations:** College of Veterinary Medicine, Northwest A&F University, Yangling, Chinaa; Department of Veterinary Medicine, National Chung Hsing University, Taichung, Taiwanb; Department of Veterinary Medicine, National Pingtung University of Science and Technology, Pingtung, Taiwanc; Institute of Molecular Biology, National Chung Hsing University, Taichung, Taiwand; Agricultural Biotechnology Center, National Chung Hsing University, Taichung, Taiwane

## Abstract

The complete genomic sequence of a new H9N2 avian influenza virus (AIV), isolated in northwestern China, was determined. Sequence and phylogenetic analyses based on the sequences of eight genomic segments revealed that the isolate is phylogenetically related to the Y280-like sublineage.

## GENOME ANNOUNCEMENT

During the last decade, H9N2 avian influenza viruses (AIVs) have circulated worldwide in poultry and caused economic losses in the poultry industry in many countries (1, 2). Asian H9N2 AIVs have been grouped into three sublineages: G1-like, Y280-like, and Y439-like (3, 4). In China, H9N2 AIVs were first isolated from Guangdong province in 1994. After that, similar viruses were isolated in many provinces and these have been the most prevalent subtype in chicken flocks (5, 6, 7, 8). Furthermore, human infection with H9N2 AIVs was reported in Hong Kong and China in 1999 and 2003, respectively (9, 10). The viruses have also been isolated from pigs in Hong Kong and China (11, 12). Shaanxi is the major poultry industry province in northwestern China. Therefore, understanding the evolution of AIVs in this area is greatly important to the prevention of AI in China.

In this study, the full-length sequences of eight genomic segments of a new H9N2 AIV isolate, A/chicken/Shaanxi/11/2012 (H9N2), were determined. Sequence and phylogenetic analyses indicated that the isolate is phylogenetically related to the Y280-like sublineage. In the present study, we found that the hemagglutinin (HA), neuraminidase (NA), and nonstructural (NS) genes of the new isolate share 92 to 98.0% nucleotide homologies with those of the H9N2 AIVs. More importantly, the nucleoprotein (NP) gene is closely related to that of the H5N1 AIVs isolated in eastern China. The matrix (M) gene has 92.0 to 93.1% nucleotide similarity with a H6N1 virus isolated from Guangzhou province in 2004, indicating that complicated reassortments of AIVs occurred in China.

The^335^RSSRGLF^341^ sequences at the cleavage site in the HA protein indicate that the isolate is a low-pathogenic type (LPAIV) (13). Seven potential glycosylation sites were observed in the HA protein. Compared with the Y280-like AIVs, a potential glycosylation site at amino acid residue 313 was seen in the new isolate, suggesting this mutation may affect virus-induced cell fusion and its receptor binding ability (14). Two amino acid substitutions at residues 191 and 198 in the receptor-binding site (RBS) in HA1 are seen in the new isolate (15). H9N2 AIV isolates contain 226L in the receptor-binding site of HA, which is typical of human H2 and H3 viruses (16). Interestingly, a L226N substitution in HA was found in the new isolate.

The new isolate does not contain the mutation E62K or D701N (17) in the RNA polymerase subunit B2 (PB2) protein, so it still retains avian virus characteristics. Furthermore, there are no amino acid substitutions in 26L, 27V, 30A, 31S, 34G, 37H, or 41W in the M protein, indicating that the new isolate is sensitive to amantadine drugs (18). It is worth noting that there is an N383D substitution at residue 383 in the RNA polymerase subunit A (PA) protein, which may increase the virulence of the virus (19). In the present study, a substitution of V149A was found in the NS1 of the new isolate, implying that this may affect the ability of viruses to antagonize interferon induction in chicken embryonic fibroblasts (20).

### Nucleotide sequence accession numbers.

The GenBank accession numbers of the new H9N2 AIV isolate from Shaanxi province are shown in Table 1.

**TABLE 1:** Nucleotide sequence accession no. of the new H9N2 AIV strain isolated from Shaanxi province in China

Isolate	PB2	PB1	PA	HA	NP	NA	M	NS
A/chicken/Shaanxi/11/2012(H9N2)	KC767264	KC767257	KC767258	KC767259	KC767260	KC767261	KC767262	KC767263
